# Effects of Management Related Practices on Turkey Hen Performance Supplemented With Either Original XPC™ or AviCare™

**DOI:** 10.3389/fvets.2018.00185

**Published:** 2018-08-13

**Authors:** Brooke M. Bartz, Don R. McIntyre, Jesse L. Grimes

**Affiliations:** ^1^Prestage Department of Poultry Science, North Carolina State University, Raleigh, NC, United States; ^2^Research & Tech Service, Diamond V, Cedar Rapids, IA, United States

**Keywords:** Turkey (*Meleagris gallapavo*), corticosterone, stress, performance, fermentation derived functional metabolites

## Abstract

During commercial production, turkeys may be exposed to several different types of stressors related to environmental conditions and management practices. Historically, antibiotics have been used to aid in the alleviation of the impacts of stressful situations, and alternatives to antibiotics related to reducing stress are being researched. This study consisted of three rearing trials and had two objectives: (1) Investigate the effects of common U.S. turkey production practices, including environmental and management induced stress, on turkey hens grown to 63 days, and (2) Determine the response of stressed birds to dietary supplementation with fermentation derived functional metabolites. Treatments consisted of a positive control (stressed but not supplemented), a negative control (non-stressed and not supplemented) and two treatment groups that were stressed and supplemented with different products (Original XPC™ or AviCare™). Supplemental products were administered in a dry (feed) and liquid (drinking water) form, and consisted of fermentation derived functional metabolites. Products were evaluated on their effectiveness to mitigate stress responses and turkey performance. Birds were exposed to some combinations of the following stressors: feed and water withdrawal, cold, heat, and crowding. Although the stressors in this study were applied for less than 24 h, they produced significant physiological responses. Blood corticosterone levels, measured immediately after stress, were significantly higher in positive control birds than in negative control birds. In addition, stressed birds had reduced body weights and increased FCR after stress. Stressed birds, supplemented with products had mixed, but generally positive response as measured by bird performance. Based on the response to stress, as observed by alterations in blood corticosterone levels and differences in performance between the two control groups, researchers induced an effective stress model. The use of supplementary products consisting of fermentation derived functional metabolites shows promise for reducing negative impacts of a mixture of stressors.

## Introduction

The stress response is a complex physiological event that involves numerous biological systems within the body. The general adaptation syndrome (GAS) outlines and discusses how an individual may respond to stressful stimuli (1). The GAS system considers the sum of all non-specific, systemic reactions of the body, which ensue upon long continued exposure to stress (1). Stress causes the nervous system to activate the “fight-or-flight” response, which is driven by catecholamines, such as epinephrine, norepinephrine, and dopamine (2). Environmental factors, transportation processes, social interactions, restricted feeding schedules, and immune system stimulation have all induced some level of stress response in poultry (3–6). In response to stress, birds may exhibit decreased growth performance, a decreased livability, alterations to immune system function, and decreased reproductive performance. Stressful conditions may lead to the production of stress hormones, such as corticosteroids, which exert direct effects on the immune system (2). Antibiotics and antimicrobials have been used to aid birds during stress to improve bird health and performance by decreasing or altering the bacterial populations present in the digestive tract (7–9). However, as consumers become more conscientious of their food products and as animal welfare concerns continue to increase, alternatives to antibiotics must be explored.

Natural feed additives with immunomodulatory properties have been explored as alternatives to antibiotics (10). Use of *Saccharomyces cerevisiae* fermentation derived products has demonstrated positive results by aiding in the alleviation of types of stress which impact poultry. Adding components derived from the fermentation process to poultry diets have been used to improve growth and impacts the physiology, morphology, and microbiology of the intestinal tract in turkeys and chickens (11, 12).

The immunomodulatory properties of fermentation derived products may serve as alternatives to antibiotics for both growth promotion, disease resistance, and food safety in poultry production (4, 11, 13–16). Fermentation derived functional metabolites (Original XPC™, Diamond V, Cedar Rapids, IA, USA) added directly in the feed, as a dry product, has been reported to improve poultry performance and health through modulation of the immune system by increased production of antibodies, increased secretory IgA and intestinal IgM, enhanced serum lysozyme activity, increased T-lymphocytes, and decreased heterophil:lymphocyte ratios (4, 14, 17–19). Feeding fermentation derived products to broilers and turkeys has also been reported to reduce the prevalence and numbers of *Salmonella, Campylobacter*, and *Escherichia coli* recovered from the gut, including a reduction in the antibiotic resistance of recovered *Salmonella* and *E. coli* colonies (13, 15, 16, 20–22). Liquid supplementation products (AviCare™, Diamond V, Cedar Rapids, IA, USA) contain fermentation derived functional metabolites which can be administered in poultry drinking water and serve as an alternative source outside of adding supplementation products to the feed.

The objectives of the study reported herein were to evaluate: (1) Effects of U.S. management practices and environmental conditions on the performance and stress response of turkey hens grown to 63 days; and (2) The ability of supplementation with fermentation derived functional metabolite products (Original XPC™ and AviCare™) to mitigate impacts of stress on bird performance. It is hypothesized that the applied management and environmental stressors, lasting less than 24 h, will have a negative effect on bird performance and the supplementation of fermentation derived functional metabolites will alleviate the stress response and improve production performance.

## Materials and methods

### Animal use protocol

This study was conducted under the supervision guidelines set forth by the Institutional Animal Care and Use Committee at North Carolina State University. All husbandry, bird well-being, and euthanasia procedures were performed with full consideration of animal welfare.

### Animal husbandry and experimental design

Three trials were conducted at the North Carolina State University's Talley Turkey Education Unit (Raleigh, NC). Each trial consisted of 960, day of hatch, large white commercial turkey hens (Nicholas Select, Aviagen Turkeys Inc., Lewisburg, WV, USA) reared to 63 days. Birds were randomly placed in 48 pens (2.44 m × 3.6 m) in a curtain-sided, naturally ventilated facility containing 12 replicate pens per treatment. Pens were divided into 4 blocks and pen assignment within block was generated using a random numbers table. In each trial, previously used litter was mixed with new pine shavings, consistent with typical U.S. poultry industry management practices, and was used as a potential challenge with microorganisms present in the previously used litter. The negative control treatment pens were bedded with fresh pine shavings. In all treatments, birds were reared under natural daylength.

In Trial 1 (May to August) and Trial 2 (December to February), birds were assigned to one of four treatments: (1) negative control—non-stressed and not supplemented (NC), (2) positive control—stressed and not supplemented (PC), (3) stressed and supplemented with a liquid product in the drinking water only (AviCare™, Diamond V, Cedar Rapids, IA, USA) (AV), and (4) stressed and supplemented with a combination of a dry dietary additive product in the feed (Original XPC™, Diamond V, Cedar Rapids, IA, USA) and a liquid product in the water (AVX). In Trial 3 (June to August) birds were assigned to one of four treatment groups: (1) negative control—non-stressed and not supplemented (NC), (2) positive control—stressed and not supplemented (PC), (3) stressed and supplemented with a dry dietary additive product in the feed (XPC), and (4) stressed and supplemented with a combination of a dry dietary additive product in the feed and a liquid product in the water (AVX). Dosage of the liquid supplementation product was determined by an earlier pilot study (unpublished). Supplementation with the liquid product was provided at an inclusion level of 160 mL/100 L from placement (0 day) to 10 and 7 days before and after the applied management stresses outlined below. The dry product was continuously provided in the diet at an inclusion level of 1.25 kg/MT. Birds were fed common basal diets consisting of a typical industry corn and soybean meal starter 1 (0–28 days) and starter 2 (28–63 days).

### Management induced stress practices

Birds in each trial were exposed to a combination of typical management and environmental stressors that are common in the U.S. turkey industry. With the exception of the NC treatment, all birds were sprayed with a gel containing a live coccidiosis vaccine (Immuncox® T, CEVA Animal Health, USA) at day of hatch and were allowed to consume the vaccine containing gel for 2 h before placement. They were also exposed to a 12 h (Trial 1) or 18 h (Trials 2 and 3) feed and water withdrawal at 34 days of age to mimic industry conditions that can occur as birds are moved from brooding to grow-out facilities. In Trial 2, an 18 h cold stress was applied at the same time as the feed and water withdrawal. This is a typical environmental stress that young commercial turkeys may experience during movement in cold weather, especially when heaters are not provided in grow-out facilities. To induce the cold stress, heat lamps were removed from the targeted pens, and building temperature was reduced from 21 to 10°C. To prevent cold stressing birds in the NC treatment, NC pens were provided with 4 heat lamps, cardboard brooding rings were placed around the perimeter of the pen, and feed and water were provided *ad libitum*. In Trial 3, in addition to the feed and water withdrawal for 18 h, a heat and crowding stress was applied for 6 h during the warmest part of the day at 34 days. This induced stress was meant to represent cooping and transport from brooding to grow-out facilities during summer seasons in warm climates. To induce this stress, birds were restricted to one corner of each pen in an area, similar to dimensions of typical commercial transport coops (0.028 m^2^/bird), and a heat lamp was placed over the birds for 6 h. For the NC treatment, no alterations were made to induce heat stress, crowding, or the addition of heat lamps. In addition, birds in the NC treatment were provided feed and water *ad libitum* during the stress induction period.

### Corticosterone blood collection

In all three trials, the effects of applied management and environmental stresses were evaluated using blood serum or plasma for the stress hormone, corticosterone (CORT). Blood was collected from the brachial wing vein, also known as the cutaneous ulnar vein, of 3 birds per pen (*n* = 144) at 35 (day of stress), 36, 42, and 63 days in Trial 1 and at 34 days (day of stress) in Trials 2 and 3. After samples were collected, sample tubes were covered with Parafilm™ and refrigerated at 4°C for 2 h for serum (Trials 1 and 2) or plasma (Trial 3) retrieval. In the case of serum, samples were rimmed using a microspatula to gently dislodge the clotted sample from the inside of each tube before centrifugation at 500 x g under refrigeration at 4°C for 30 min. This added step before centrifugation was necessary, since turkey blood has added clotting factors which can cause the serum to stick under a clot formed at the top of the glass tube making it difficult to obtain the serum sample. This complication was the reason for the switch to analyzing plasma samples in Trial 3; there was a better opportunity for plasma retrieval at this bird age compared to serum. After centrifugation, samples were placed in −20°C for short term storage to ensure proper freezing and then transferred to −80°C for long term storage. Hormone levels in samples were quantified using a corticosterone ELISA kit (Cayman Chemical, Ann Arbor, MI, USA; Item: 501320). Scanes (23) reported that, with avian species, results of glucocorticoid analysis can be highly variable and inconsistent among researchers and commercially available assay kits. Therefore, sample aliquots were run using two different kit protocols: one without extraction of CORT in which samples were run without any manipulation, and a second set, in which CORT was extracted from the serum or plasma prior to analysis. For extraction, equipment used included: Extrelut NT1 columns (MilliporeSigma Item: 1.15094.0001), a 9:1 ratio of methylene chloride:propanol mixture, and evaporation occurred under a slow, steady stream of nitrogen to separate the high lipid content or cross reactivity within the samples. Samples were reconstituted in kit ELISA buffer prior to performing the assay and stored at −20°C until quantification. The reason for analyzing both unextracted and extracted samples is because the ELISA kit stated that extraction was optional, however recommended, for samples that needed additional purification due to potential cross-reactivity. In Trial 1, limited serum sample retrieval restricted analysis to unextracted samples only.

### Performance measurements

In all trials, production performance measurements included body weight (BW), feed and water intake, and mortality. Feed conversion ratio (FCR) was calculated. Bird BW was measured by pen at placement (0 day) and individual weights by pen at 34, 35, 42, and 63 days. Feed intake was recorded on a per pen basis using a tube feeder and water intake (monitored during liquid supplementation periods) was recorded on a per pen basis by weight using jug drinkers. Empty feeder weights were recorded, and any addition of feed was recorded on a pen sheet provided on each pen door. In the occurrence of any mortality or switching phases of feed type, feeders were weighed again to accurately measure FCR and to determine the amount of feed left at the end of each phase. Jug weights were recorded at the beginning of each supplementation phase to determine the weight of the empty jug and weighed again once water was added. When water levels were low, but not completely empty, the jugs were weighed again to calculate the difference in starting and finishing weight. During all other periods, water was provided by a bell-type drinker. All dead birds were removed from pens daily, weighed, and BW recorded. FCR was calculated on a pen basis by time period and cumulatively. Corrections were made for any mortality that may have occurred during specified time increments.

## Statistical analysis

For each trial, separate linear models with additive block and diet effects were fitted using the GLM procedure within JMP 11.2 (SAS Institute, Cary, NC, USA, 2015) for ANOVA. Pairwise comparisons among treatments were conducted using Tukey's procedure to control the experimentwise type 1 error using the LSMeans procedure within JMP and significance was recognized at *P* ≤ 0.05, unless otherwise stated. For all corticosterone data analysis, body weight, block, and treatment effects were added as covariates to control for any variation of corticosterone level related to body weight.

## Results

In all three trials, no differences were observed between treatments for livability or water consumption and no mortality occurred during the stress induction time periods (data not shown). There were no differences observed by treatment in any of the three trials for placement BW (0 day).

### Trial 1: feed and water withdrawal stress

The unextracted serum CORT data for Trial 1 are presented in Table 1. On the day of stress, the CORT level of the PC (stressed, not supplemented) birds was significantly higher compared to the NC (non-stressed, not supplemented) birds (*P* = 0.03). Birds that received either form of supplementation had an intermediate response in CORT compared to the control groups. The day following stress, 1 week post-stress, and at the conclusion of this trial, there were no significant differences between treatments for CORT levels. (*P* = 0.74, 0.16, and 0.16, respectively).

**Table 1:** Serum corticosterone (pg/ml) of turkey hens subjected to a 12 h fasting stress, grown to 63 days of age, and supplemented with fermentation derived functional metabolites.

**Treatment**	**35 days**	**36 days**	**42 days**	**63 days**
NC	304^b^±114	152 ± 34	128 ± 29	33 ± 11
PC	771^a^±109	198 ± 32	99 ± 30	21 ± 12
AV^1^	482^ab^±109	171 ± 34	172 ± 31	59 ± 12
AVX^1^^,^^2^	588^ab^±107	155 ± 32	189 ± 33	35 ± 12
	***P*****-value**			
	0.03	0.74	0.16	0.16

1 *AviCare (Diamond V, Cedar Rapids, IA, USA) was supplemented in the water intermittently at 160 mL/100 L*.

2 *Original XPC (Diamond V, Cedar Rapids, IA, USA) was supplemented continuously in the feed at 1.25 kg/MT*.

a, b *Values within the same column lacking the same superscript are significantly different (P ≤ 0.05)*.

Prior to the 12 h fasting stress at 34 days, BW were not significantly different (*P* = 0.16, Table 2). At 35 days, BW, following the 12 h feed and water withdrawal stress, were significantly reduced for birds in the PC, AV, and AVX treatments compared to the non-stressed birds (NC) (*P* < 0.0001). No significant differences in BW were observed between the NC, PC, AV, and AVX groups at 1 week post-stress (42 days) (*P* = 0.19). However, at 63 days, the AVX birds were heavier in BW than the PC and AV birds and the NC treatment birds were intermediate in BW (*P* = 0.005).

**Table 2:** Body weights (kg) of turkey hens subjected to a 12 h fasting stress, grown to 63 days, and supplemented with fermentation derived functional metabolites.

**Treatment**	**34 days**	**35 days**	**42 days**	**63 days**
NC	1.395 ± 0.009	1.392^a^ ± 0.008	2.053 ± 0.017	4.747^ab^ ± 0.043
PC	1.375 ± 0.009	1.268^b^ ± 0.008	2.016 ± 0.017	4.597^b^ ± 0.043
AV^1^	1.372 ± 0.009	1.258^b^ ± 0.008	2.013 ± 0.017	4.604^b^ ± 0.043
AVX^1^^,^^2^	1.393 ± 0.009	1.288^b^ ± 0.008	2.050 ± 0.017	4.783^a^ ± 0.043
	***P*****-value**			
	0.16	0.0001	0.19	0.005

1 *AviCare (Diamond V, Cedar Rapids, IA, USA) was supplemented in the water intermittently at 160 mL/100 L*.

2 *Original XPC (Diamond V, Cedar Rapids, IA, USA) was supplemented continuously in the feed at 1.25 kg/MT continuously*.

a, b *Values within the same column lacking the same superscript are significantly different (P ≤ 0.05)*.

The FCR results for Trial 1 are presented in Table 3, reported as increments of time based on applied stress, supplementation period of liquid product, and conclusion of trial. There were no significant differences between treatments for FCR on both the day before the 12 h fasting stress (34 days) and the day after (36 days). However, at 1 week post-stress (42 days), NC birds had significantly better FCR than PC birds (*P* = 0.05, Table 3). Birds that received a combination of the liquid and dry supplements (AVX) had a FCR similar to the NC birds and birds that received only the liquid supplement (AV) had an intermediate FCR. Results for FCR from the day of stress to 1 week subsequent (34–42 days) were similar (data not shown). However, there were no significant differences in FCR at 63 days due to treatments (*P* = 0.62).

**Table 3:** Feed conversion ratio (kg Feed: kg Gain) of turkey hens subjected to a 12 h fasting stress, grown to 63 days, and supplemented with fermentation derived functional metabolites.

**Treatment**	**0–34 days**	**0–42 days**	**0–63 days**
NC	1.518 ± 0.012	1.641^b^±0.011	1.660 ± 0.009
PC	1.527 ± 0.012	1.672^a^±0.011	1.658 ± 0.009
AV^1^	1.532 ± 0.012	1.660^ab^±0.011	1.661 ± 0.009
AVX^1^^,^^2^	1.525 ± 0.012	1.630^b^±0.012	1.646 ± 0.009
	***P*****-value**		
	0.87	0.05	0.62

1 *AviCare (Diamond V, Cedar Rapids, IA, USA) was supplemented in the water intermittently at 160 mL/100 L*.

2 *Original XPC (Diamond V, Cedar Rapids, IA, USA) was supplemented continuously in the feed at 1.25 kg/MT*.

a, b *Values within the same column lacking the same superscript are significantly different (P ≤ 0.05)*.

### Trial 2: feed and water withdrawal stress + cold stress

Based on unextracted serum CORT measurements obtained after the 18 h cold stress, there was an increase in CORT when comparing the NC (non-stressed, not supplemented) treatment birds to the PC birds (stressed, not supplemented) treatment with a *P*-value approaching significance (*P* = 0.10, Table 4). Birds that were fed the supplemental products (AV and AVX) were intermediate in response (Table 4). Responses measured in the extracted CORT samples were similar in PC birds with a significant increase in serum corticosterone compared to NC birds (*P* = 0.0001). Birds that received the liquid supplement (AV) were intermediate in response, whereas the birds that received a combination of products (AVX) were not significantly different than the PC birds.

**Table 4:** Serum corticosterone levels (pg/ml) following an 18 h fasting and cold stress in turkey hens, grown to 63 days, and supplemented with fermentation derived metabolite products.

	**Pre-extracted**	**Post-extracted**
**Treatment**	**34 days**	**34 days**
NC	49^B^±56	5112^b^±1419
PC	274^A^±63	10893^a^1±1309
AV^1^	135^AB^±64	9593^ab^±1319
AVX^1^^,^ ^2^	181^AB^±56	10788^a^±1256
*P*-value	0.10	0.0001

1 *AviCare (Diamond V, Cedar Rapids, IA, USA) was supplemented in the water*.

2 *Original XPC (Diamond V, Cedar Rapids, IA, USA) was supplemented continuously in the feed at 1.25 kg/MT*.

A, B *Values within the same column lacking the same superscript are significantly different (P ≤ 0.10)*.

a, b *Values within the same column lacking the same superscript are significantly different (P ≤ 0.05)*.

At 34 days, prior to the application of the 18 h fasting and applied cold stress, birds in the AVX treatment were heavier in BW than birds in the NC, PC, and AV treatments (*P* = 0.005, Table 5). At 35 days, immediately following the applied stress, birds in the NC treatment were significantly heavier than birds in all other treatment groups (PC, AV, and AVX) (*P* < 0.0001) and birds in the AVX treatment were significantly heavier than the PC and AV birds at 35 days (*P* < 0.0001). At 42 days, 1 week post the applied stress, the birds in the AVX group were heavier in BW than the birds in the PC and AV treatments; birds in the NC treatment were now intermediate in BW (*P* = 0.0003). At 63 days, the BW differences among treatments were no longer significant, but the same pattern in BW across treatments was evident (*P* = 0.08).

**Table 5:** Body weights (kg) of turkey hens subjected to an 18 h fasting and cold stress, grown to 63 days, and supplemented with fermentation derived functional metabolites.

**Treatment**	**34 days**	**35 days**	**42 days**	**63 days**
NC	1.645^b^ ± 0.015	1.716^a^ ± 0.013	2.324^ab^ ± 0.016	5.383 ± 0.031
PC	1.634^b^ ± 0.015	1.530^c^ ± 0.013	2.268^b^ ± 0.016	5.346 ± 0.033
AV^1^	1.648^b^ ± 0.015	1.540^c^ ± 0.013	2.278^b^ ± 0.016	5.297 ± 0.031
AVX^1^^,^ ^2^	1.706^a^ ± 0.015	1.609^b^ ± 0.013	2.367^a^ ± 0.016	5.415 ± 0.035
	***P*****-value**			
	0.005	< 0.0001	0.0003	0.08

1 *AviCare (Diamond V, Cedar Rapids, IA, USA) was supplemented in the water intermittently at 160 mL/100 L*.

2 *Original XPC (Diamond V, Cedar Rapids, IA, USA) was supplemented continuously in the feed at 1.25 kg/MT*.

a, b, c *Values within the same column lacking the same superscript are significantly different (P ≤ 0.05)*.

The FCR for Trial 2 are presented in Table 6. There were no differences in FCR between the treatment groups leading up to the 18 h fasting and cold stress at 34 days (*P* = 0.47). Similar results were observed at 42 days, 1 week post-stress (*P* = 0.19) and there were no differences due to treatment at 63 days (*P* = 0.45).

**Table 6:** Feed conversion ratio (kg Feed: kg Gain) of turkey hens subjected to an 18 h fasting and cold stress, grown to 63 days, and supplemented with fermentation derived functional metabolites.

**Treatment**	**0–34 days**	**0–42 days**	**0–63 days**
NC	1.412 ± 0.014	1.454 ± 0.009	1.606 ± 0.010
PC	1.411 ± 0.014	1.477 ± 0.009	1.617 ± 0.011
AV^1^	1.414 ± 0.014	1.478 ± 0.009	1.598 ± 0.010
AVX^1^^,^ ^2^	1.386 ± 0.014	1.459 ± 0.009	1.618 ± 0.010
	***P*****-value**		
	0.47	0.19	0.45

1 *AviCare (Diamond V, Cedar Rapids, IA, USA) was supplemented in the water intermittently at 160 mL/100 L*.

2 *Original XPC (Diamond V, Cedar Rapids, IA, USA) was supplemented continuously in the feed at 1.25 kg/MT*.

### Trial 3: feed and water withdrawal stress + heat and crowding stress

Plasma corticosterone results for Trial 3 are presented in Table 7. Based on unextracted CORT results there were no differences between treatments at 34 days (*P* = 0.34). However, there was a significant increase in extracted plasma CORT in the PC, XPC, and AVX treatment birds compared to the NC treatment birds (*P* < 0.0001).

**Table 7:** Plasma corticosterone levels (pg/ml) following an 18 h fasting and 6 h heat and crowding stress in turkey hens, grown to 62 days, and supplemented with fermentation derived functional metabolites.

	**Pre-extracted**	**Post-extracted**
**Treatment**	**34 days**	**34 days**
NC	327 ± 154	6740^b^±1138
PC	659 ± 117	12459^a^±876
XPC^2^	446 ± 111	12000^a^±876
AVX^1^^,^ ^2^	448 ± 117	14131^a^±831
*P*-value	0.34	0.0001

1 *AviCare (Diamond V, Cedar Rapids, IA, USA) was supplemented in the water intermittently at 160 mL/100 L*.

2 *Original XPC (Diamond V, Cedar Rapids, IA, USA) was supplemented continuously in the feed at 1.25 kg/MT*.

a, b *Values within the same column lacking the same superscript significantly different (P ≤ 0.05)*.

At 34 days, prior to 18 h fasting and 6 h heat & crowding stress, NC birds were significantly heavier than PC birds and AVX treatment birds; birds in the XPC group were intermediate in BW at 34 days (*P* = 0.002, Table 8). At 35 days, the day following the stress, treatment birds that received the fasting stress were significantly lighter in BW than NC birds (*P* < 0.0001). At 45 days, NC birds were heavier than PC birds with birds that received either form of supplementation product being intermediate in response (*P* = 0.03). At 62 days, the end of the trial, birds that were stressed at 34 days were still lower in BW than birds that were not stressed (*P* = 0.04), and stress birds that received either form of supplementation (XPC or AVX) were intermediate in response.

**Table 8:** Body weights (kg) of turkey hens subjected to an 18 h fasting and 6 h heat and crowding stress, grown to 62 days, and supplemented with fermentation derived functional metabolites.

**Treatment**	**34 days**	**35 days**	**45 days**	**62 days**
NC	1.452^a^±0.013	1.536^a^±0.014	2.218^a^±0.020	4.652^a^±0.029
PC	1.378^b^±0.013	1.253^b^±0.014	2.131^b^±0.020	4.526^b^±0.030
XPC^2^	1.413^ab^±0.013	1.283^b^±0.013	2.189^ab^, 0.020	4.602^ab^±0.027
AVX^1^^,^ ^2^	1.400^b^±0.013	1.288^b^±0.013	2.161^ab^±0.020	4.606^ab^±0.027
	***P*****-value**			
	0.002	0.0001	0.03	0.04

1 *AviCare (Diamond V, Cedar Rapids, IA, USA) was supplemented in the water intermittently at 160 mL/100 L*.

2 *Original XPC (Diamond V, Cedar Rapids, IA, USA) was supplemented continuously in the feed at 1.25 kg/MT*.

a, b *Values within the same column lacking the same superscript are significantly different (P ≤ 0.05)*.

There were no significant differences between treatments with regards to FCR immediately prior to the 18 h fasting and 6 h heat and crowding stress at 34 days (*P* = 0.96, Table 9). On the day of stress, birds in all treatments that received the fasting stress had a significantly better FCR than NC birds (data not shown). At 45 days there were no differences in FCR between any of the treatment groups (*P* = 0.99). At 63 days, 4 weeks post-stress, there was a trend (*P* = 0.11) for cumulative FCR to be improved for birds in the AVX group (1.601 ± 0.008) compared to birds in the NC (1.629 ± 0.008) and PC groups (1.623 ± 0.008).

**Table 9:** Feed conversion ratio (kg Feed; kg Gain) of turkey hens subjected to an 18 h fasting and 6 h heat and crowding stress, grown to 62 days, and supplemented with fermentation derived functional metabolites.

**Treatment**	**0–34 days**	**0–45 days**	**0–62 days**
NC	1.520 ± 0.016	1.563 ± 0.012	1.629 ± 0.008
PC	1.528 ± 0.016	1.564 ± 0.012	1.623 ± 0.008
XPC^2^	1.532 ± 0.016	1.563 ± 0.012	1.606 ± 0.008
AVX^1^^,^ ^2^	1.528 ± 0.016	1.563 ± 0.012	1.613 ± 0.008
	***P*****-value**		
	0.96	0.99	0.11

1 *AviCare (Diamond V, Cedar Rapids, IA, USA) was supplemented in the water intermittently at 160 mL/100 L*.

2 *Original XPC (Diamond V, Cedar Rapids, IA, USA) was supplemented continuously in the feed at 1.25 kg/MT*.

## Discussion

The poultry industry is important for economic growth and food production in several countries, and exposure of birds to stressful conditions, disease challenges, and deterioration of environmental conditions may result in serious economic loss (8). St-Pierre et al. (24) reported a $2.4 billion U.S. annual economic loss across the U.S. livestock industry, including turkeys, due to heat stress where livestock are reared under conditions outside the animal's thermo-neutral zone. These losses included increased mortality and a decreased performance and reproduction. Losses were estimated to occur even with the use of heat abatement practices. Early life experiences may influence interactions with the environment and lead to a varied response in health, welfare, and productivity (25). Stress can be physical, chemical, or physiological in nature (26) and individual animals differ in their reactivity to stress and fearfulness (27). Therefore, it may be hypothesized that birds which have difficulty maintaining homeostasis may be at greater risk for disease contraction and production losses.

Of the two main objectives tested in this study the first was to determine the effect of management applications of common U.S. turkey industry management practices and environmental conditions on performance and stress response in turkey hens grown to 63 days. Subjecting birds to common environmental stressors at 34 days (feed and water withdrawal, cold stress, and heat and crowding stress) produced, in all cases, alterations in bird performance and blood corticosterone response. While stressors were temporary (less than 24 h), the responses by turkeys were both immediate, as measured by increases in corticosterone levels, and long term, with differences in performance observed and measured over several days. The return of the PC birds' performance levels to those observed in the NC birds was not surprising, given the short duration stressors were applied. Selye (1) described the principles behind the GAS system in which the end goal is to return to physiological homeostatic conditions within the individual as represented by the NC treatment in this present work. While the birds described herein were exposed to stressors for short durations, birds in field conditions might have longer durations of stressor exposure, which may have continued depressed performance as observed by decreases in PC treatments in the current study.

Corticosterone is the major adrenal glucocorticoid in birds, and is important in the regulation of metabolic, cardiovascular, immune, and behavioral processes (28). Secretion increases when the hypothalamic-pituitary-adrenal axis is activated in response to a stressor (29). Corticosterone has a daily cycle (30) and it has been documented that the half-life of this glucocorticoid in Japanese quail is approximately 10–15 min (29). It is highly variable in other poultry species depending on the type of assay used for determination and methodology followed in kit guidelines (23). Lendvai et al. (31) reported that house sparrow plasma corticosterone levels are negatively associated with changes in body mass; loss of body mass was associated with increases in corticosterone. Based on both the unextracted and extracted corticosterone measurements in these three trials, turkeys responded with increased blood corticosterone levels (pg/mL) when exposed to stressors. The fact that differences in corticosterone levels were no longer significant following the initial stress response (36 days) was expected, because birds were physiologically returning to homeostatic conditions. El-Halawani et al. (30) exposed 9 wk old male turkeys to either high or low temperatures of 32°C or 7°C compared to a control group held at 24°C for 24 h. The plasma corticosterone level increased for both temperature stressed groups and then returned to the control group levels within the third week of the trial, similar to results of the present study. The decrease in bird performance following the application of three different stressors that was observed in this study was similar to results from other studies. Huff et al. (6) stressed male and female turkeys during a rearing trial at 6, 12, and 16 weeks of age with transport, holding birds for 9 h without feed and water, and subjecting them to an *E. coli* challenge. Challenged birds experienced decreased BW and increased FCR compared to controls. There was some recovering in BW of the stressed birds in between weeks of stress application. Boukhris et al. (32) applied transport stresses up to 150 min in 16 weeks old male turkeys to simulate different transport times to processing destinations. Those researchers observed that long-term transport had negative effects on post-mortem meat quality and concluded that transport stress may induce pale, soft, and exudative meat. The same might have been true in the current study, however, analysis of post-mortem meat characteristics were not conducted. The genetics of turkeys can affect their response to stress. Huff et al. (33) challenged three genetics lines of male and female turkeys with stressors including transport and holding times. The genetic lines selected for increased BW were observed to have increased negative responses to the stressors compared to a line that was selected for egg production. In another study, Ognik and Krauze (34) reported that a dietary supplement with of a *S. cerevisiae* product that served as a prebiotic additive in the diet of turkey hens grown to 6 wk had influences on other blood chemistry markers, including an increase in antioxidant parameters leading to improved growth performance. Similar improvements and intermediary responses were observed in the current study.

Nutrient and non-nutrient components of the diet can impact the development, maintenance, and response of the immune system (2) and it has been observed that addition of *S. cerevisiae* fermentation, its fractions, and other metabolites harvested during the fermentation process, have the potential to alleviate stress and minimize effects of unfavorable conditions in poultry production (11, 35). In the present study, the addition of fermentation derived functional metabolites to the diet of turkeys improved bird performance as observed by improvements in BW after induced stress. Huff et al. (36) fed extracts from *S. cerevisiae* to male and female turkeys to determine whether products would improve turkey performance following their exposure to stress. Stress challenges included: dexamethasone injection, environmental exposure to *E. coli* and *Listeria monocytogenes*, and transport stress (12 h with holding time). Stressed poults fed the *S. cerevisiae* extract in their study had increased BW and BW gain and decreased mortality compared to unsupplemented poults. Transport did not affect BW or serum corticosterone. In contrast, Dexamethasone decreased BW; however, when exposed birds received *S. cerevisiae* extract, serum corticosterone decreased. Firman et al. (18) measured an improvement of FCR in male turkeys when supplemented with similar treatments in feed. Price et al. (37) reared broilers under heat stress conditions from 28 to 42 days via daily temperature raised from control level of 24–35°C for 18 h. In their study, rearing under heat stress conditions resulted in increased plasma corticosterone, but stressed birds receiving feed treatment, similar to those used herein, resulted in reduced plasma corticosterone. Furthermore, Al-Mansour et al. (17) noted an improvement in FCR in chicks receiving a *S. cerevisiae* derived product. Similar improvements were observed in these trials following management induced stress. Since *S. cerevisiae* derived products have metabolites, peptides, organic acids, oligosaccharides, and some potential growth factors associated with dietary supplementation (4), these fermentation products may aid the birds during management induced stress periods.

## Conclusion

In conclusion, management applied stressors, such as feed and water withdrawal, cold, heat, or crowding, can induce stress resulting in an increase in measurable blood corticosterone levels and negatively affect commercial turkey hen production performance. However, supplementation with the fermentation derived functional metabolites found in dry (Original XPC™) and liquid (AviCare™) commercially available products may be able to partially alleviate the stress response in turkey hens in the period of time following the stress. The cost effectiveness of using the fermentation derived functional metabolites products used in this study must be determined by the end user based on their own production parameters, conditions, and economics.

## Author contributions

BB was project lead for this research and wrote the majority of this manuscript under the direction of JG's lab. DM supplied the products used in this body of research to be tested and aided in final edits of manuscript. JG was in charge of the laboratory responsible for this body of research, arranged logistics, and contributed ideas and edits to the final manuscript.

### Conflict of interest statement

The authors declare that the research was conducted in the absence of any commercial or financial relationships that could be construed as a potential conflict of interest.
